# Ser96Ala genetic variant of the human histidine-rich calcium-binding protein is a genetic predictor of recurrence after catheter ablation in patients with paroxysmal atrial fibrillation

**DOI:** 10.1371/journal.pone.0213208

**Published:** 2019-03-06

**Authors:** Michitaka Amioka, Yukiko Nakano, Hidenori Ochi, Yuko Onohara, Akinori Sairaku, Takehito Tokuyama, Chikaaki Motoda, Hiroya Matsumura, Shunsuke Tomomori, Naoya Hironobe, Yousaku Okubo, Sho Okamura, Kazuaki Chayama, Yasuki Kihara

**Affiliations:** 1 Department of Cardiovascular Medicine, Hiroshima University Graduate School of Biomedical and Health Sciences, Hiroshima, Japan; 2 Chuden Hospital, Hiroshima, Japan; 3 Department of Cellular and Molecular Biology, Graduate School of Biomedical Science, Hiroshima University, Hiroshima, Japan; Indiana University, UNITED STATES

## Abstract

**Background:**

Atrial fibrillation (AF) recurrence after radiofrequency catheter ablation (RFCA) still remains a serious issue. Ca^2+^ handling has a considerable effect on AF recurrence. The histidine-rich calcium-binding protein (*HRC*) genetic single nucleotide polymorphism (SNP), rs3745297 (T>G, Ser96Ala), is known to cause a sarcoplasmic reticulum Ca^2+^ leak. We investigated the association between *HRC* Ser96Ala and AF recurrence after RFCA in paroxysmal AF (PAF) patients.

**Methods and results:**

We enrolled PAF patients who underwent RFCA (*N* = 334 for screening and *N* = 245 for replication) and were genotyped for *HRC* SNP (rs3745297). The patient age was younger and rate of diabetes and hypertension lower in the PAF patients with Ser96Ala than in those without (TT/TG/GG, 179/120/35; 64±10/60±12/59±13 y, *P* = 0.001; 18.5/ 9.2/8.6%, *P* = 0.04 and 66.1/50.0/37.1%, *P* = 0.001, respectively). During a mean 19 month follow-up, 57 (17.1%) patients suffered from AF recurrences. The rate of an Ser96Ala was significantly higher in patients with AF recurrence than in those without in the screening set (allele frequency model: odds ratio [OR], 1.80; *P* = 0.006). We also confirmed this significant association in the replication set (OR 1.74; *P* = 0.03) and combination (*P* = 0.0008). A multivariate analysis revealed that the AF duration, sinus node dysfunction, and *HRC* Ser96Ala were independent predictors of an AF recurrence (hazard ratio [HR], 1.04, *P* = 0.037; HR 2.42, *P* = 0.018; and HR 2.66, *P* = 0.007, respectively).

**Conclusion:**

*HRC* SNP Ser96Ala is important as a new genetic marker of AF recurrence after RFCA.

## Introduction

Pulmonary vein (PV) isolation has been an established ablation technique for curing paroxysmal atrial fibrillation (PAF). However, approximately 13 to 39% of patients demonstrate recurrent PAF after radiofrequency catheter ablation (RFCA) [1]. Several factors, such as the duration of atrial fibrillation (AF), hypertension, diabetes, sleep apnea, obesity, PV reconnections, initiation from non-PV foci, and atrial remodeling, have been reported to be contributing factors to AF recurrence [2–5]. Genetic variants of the AF-related gene (*PITX2*) also have been reported to be associated with AF recurrence, but this remains controversial [6].

A recent meta-analysis revealed that even among AF-free patients, 58.6% had at least one electrically reconnected PV. This result suggested that PV reconnections do not always lead to AF recurrence and whether to factor AF recurrence out of PAF patients with PV reconnections has not been defined [7]. Abnormal Ca^2+^ handling has been known to evoke PV triggers. The regulatory proteins of Ca^2+^ handling, including protein kinase A, calmodulin kinase II, phospholamban, and ryanodine receptor type 2, are important contributors to the sarcoplasmic reticulum (SR) Ca^2+^ overload and diastolic membrane instability [8, 9]. The histidine-rich calcium-binding protein (*HRC*) is also known as the key modulator of Ca^2+^ handling in the SR [10–13]. The *HRC* is a member of the SR Ca^2+^ release channel complex and has an important role in Ca^2+^ uptake regulation via a direct interaction [13–15].

The human genetic variant of the *HRC*, rs3745297 (Ser96Ala), impairs the function of *HRC* binding to triadin, attenuates the inhibitory effect on the Ca^2+^ release via ryanodine receptors, and results in a Ca^2+^ overload [13, 16]. An experimental study with adult rats showed that acute overexpression of the human Ala96 *HRC* variant in their cardiomyocytes by an adenoviral gene transfer increased the sarcoplasmic reticulum Ca^2+^ leak and frequency of Ca^2+^ sparks [17]. Ser96Ala has been reported to cause life-threatening ventricular arrhythmias and sudden death in patients with idiopathic dilated cardiomyopathy [18, 19]. The association between Ser96Ala and ventricular arrhythmias has not been reported in normal structural hearts. Han et al. expressed the human wild-type and Ser96Ala variant of *HRC* in normal or heart failure rat myocytes. The *HRC* 96Ala increased the frequency of spontaneous Ca^2+^ sparks and a higher SR Ca^2+^ load. Their results suggested that *HRC* Ser96Ala modulated the HRC function also in normal cardiomyocytes [20]. The HRC has been reported to be distributed also on the atrium in mammals, however, the expression level differs depending on the animal species [21].

We hypothesized that Ser96Ala was related to the AF recurrence after RFCA in patients with PAF and investigated the relationship between Ser96Ala and AF recurrence.

## Methods

### Participants

We included 382 consecutive Japanese patients with PAF who had undergone an initial RFCA between September 2011 and February 2014 at Hiroshima University Hospital for screening. Among them, 48 patients were excluded because they had been lost to follow-up within 1 year after the RFCA. Ultimately, we enrolled 334 Japanese PAF patients (256 men, 78 women; mean age, 62 ± 11 years) as a screening cohort. We also enrolled 245 consecutive Japanese PAF patients (174 men, 71 women; mean age, 66 ± 10 years) who underwent catheter ablation at Hiroshima University Hospital between March 2014 and September 2016 for replication. The Institutional Ethics Committee of the Graduate School of Biomedical Science at Hiroshima University approved all procedures involving human genome use. Written informed consent was obtained from all participants.

### Echocardiography

Complete echocardiographic studies (transthoracic and transesophageal echocardiography) were performed in all patients within 24 h before the RFCA using commercially available ultrasonography systems (Vivid 7; GE Healthcare, Milwaukee, WI, USA; EPIQ7; Philips Medical Systems, Andover, MA, USA; or Artida; Toshiba Medical Systems, Tochigi, Japan) by four experienced sonographers who were blinded to the clinical information. Echocardiographic measurements were obtained following the recommendations of the American Society of Echocardiography.

### Electrophysiological study and RFCA

The patients were treated with warfarin or direct anticoagulants at least 1 month before the RFCA and throughout the periprocedural period. All antiarrhythmic drugs (AADs) other than amiodarone were stopped at least five half-lives before the RFCA. Amiodarone was discontinued at least 2 weeks before the RFCA. Two circular mapping catheters (Lasso; Biosense Webster, Diamond Bar, CA, USA) were positioned within the ipsilateral superior and inferior left PVs under the guidance of selective PV angiography, and the RF catheter was positioned in the right superior PV. A 6-F multipolar (15-pole) catheter (BeeAT, Japan Lifeline, Japan) was utilized with the distal poles (poles 1−8) placed within the coronary sinus and proximal electrodes (poles 9−15) located from the superior vena cava to the superior right atrium (RA) via the right internal jugular vein. A fixed multipolar electrode catheter (Abott, Cicago, USA) was introduced via the femoral vein to the His bundle and RV regions. Intravenous isoproterenol (ISP) was administered to the right femoral vein (10 μg flash) to induce AF before an extensive encircling pulmonary vein isolation (EEPVI). If AF was not induced, intravenous adenosine 5′-triphosphate (ATP) (10 mg) was administered immediately after the ISP infusion (10 μg flash). ISP was added if AF was not induced up to 30 μg. A continuous EEPVI was performed 0.5 to 2.0 cm from the PV ostia using a guided three-dimensional electroanatomical mapping system (Carto3; Biosense Webster) with computed tomography integration (Cartomerge; Biosense Webster) to achieve electrical isolation of the left and right PVs. An irrigated 3.5 mm tip electrode catheter (THERMOCOOL SmartTouch SF; Biosense Webster) was used for the EEPVI. Radiofrequency energy was delivered for 15 to 20 s at each point around the EEPVI line to achieve a reduced amplitude of the local bipolar atrial electrograms of >80% or <0.1 mV. Successful PV isolation was defined as the loss of all PV potentials (entrance block) and failure to capture the left atrium when pacing from the PV (output 5 mA, pulse width 2 ms, exit block) using a circular multipolar mapping catheter under an injection of both ISP and ATP. The EEPVI was achieved in all subjects with PAF. Within 1 h of obtaining stable sinus rhythm, an electrophysiological study was performed. Then, the AA interval (onset or peak of one atrial signal to the same aspect of the next consecutive signal), AH interval (atrial signal to His bundle), and HV interval (His bundle to the first ventricular activation) were measured. The corrected sinus node recovery time (cSNRT) was defined as the recovery interval beyond the sinus cycle (i.e., cSNRT = maximum SNRT − sinus cycle length).

### *HRC* SNP genotyping

Peripheral blood was obtained, and the genomic DNA was extracted from leukocytes using a QIAamp DNA Blood Mini Kit (Qiagen, Hilden, Germany) according to the standard protocol. We genotyped the *HRC* (rs3745297, T > G, Ser96Ala) in all participants using the TaqMan assay as described previously [22, 23]. For typing the rs3745297, we used a forward primer CCTCATCTCCGACTTTGTGGTC and reverse primer CAGCCCTAGAGACCATCCAGAT. We also used an Invader oligo GGATCTTGCATGGCCTCGTAGTGAGGTGAT, signal prove-T CGCGCCGAGGcgcgccgaggAGACATYTTCATCCTCCTTTTCcgcgccgaggGGGATTTTGTCTTCACACTAA, and signal prove-G atgacgtggcagacCGACATYTTCATCCTCCTTTT.

### Follow-up after RFCA

After the RFCA, the patients received the medications required. AADs (including classes IA, IC, II, and III and bepridil according to the Vaughan–William classification) were used to maintain sinus rhythm during the blanking period. Each patient was scheduled to be examined in the outpatient clinic at 1, 3, and 6 months and then every 6 months thereafter. Twelve-lead electrocardiography, echocardiography, and 24 h Holter monitoring were repeated at each clinical visit to assess the presence or absence of an AF recurrence. The patients were strictly instructed to be seen in the outpatient clinic or emergency department when they had palpitations or a disturbed pulse. If an abnormality was detected, we applied 24 h Holter monitoring or a portable electrocardiography monitor to detect the presence of AF. AF recurrence was defined as an episode of palpitations lasting >30 s or as AF, atrial flutter, or atrial tachycardia episodes lasting >30 s. Late AF recurrence was defined as that detected >2 months after ablation.

### Statistical analysis

The continuous variables were summarized as means ± SD and categorical variables as proportions. A Mann–Whitney *U* test was used to compare the normally distributed variables. Comparisons of paired data were performed using a Wilcoxon signed-rank test. The differences among the three genotypes were analyzed by a linear regression for continuous data. Deviation from the Hardy–Weinberg equilibrium was tested in cases and controls with the χ^2^ test. *P* values <0.05 were considered significant. Odds ratios (OR) and 95% confidence intervals (CI) were calculated for the reference allele or genotype. To test the genetic relationships between the cases and controls and evaluate the genotype distribution, we used the χ^2^ and Cochran–Armitage trend tests. For a meta-analysis of 2 individual cases and controls, we used the Mantel-Haenszel test. Kaplan–Meier analysis was used to assess the time required to achieve AF recurrence. The log-rank test was applied to compare event-free survival data between patient groups. The Cox proportional hazard model was used to determine the predictors of the end point. Variables with a value of *P* < 0.1 in the univariate models were included in the multivariate models. All statistical analyses were performed with the use of JMP software version 12.0 (SAS Institute, Cary, NC, USA). A value of *P* < 0.05 was considered statistically significant.

## Results

### Characteristics of PAF patients in each *HRC* SNP genotype

The *HRC* SNP (rs3745297, T > G, Ser96Ala) TT, TG, and GG genotypes were recognized in 179 (53.6%), 120 (35.9%), and 35 (10.5%) of the PAF patients, respectively. The minor allele frequency (MAF) of the *HRC* SNP in the PAF patients was 28.4%. The clinical characteristics of the PAF patients with each *HRC* SNP genotype is summarized in Table 1. The mean age was significantly younger in the PAF patients with a minor G allele than in those without (TT/TG/GG, 64 ± 10/60 ± 12/59 ± 13 years, respectively; *P* = 0.001). The duration of AF was similar regardless of the *HRC* SNP genotypes. Additionally, the frequencies of hypertension and diabetes mellitus and CHADS2 score were significantly lower in the PAF patients with a minor G allele than in those without: hypertension (117 [66.1%], 60 [50.0%], 13 [37.1%], *P* = 0.001); diabetes mellitus (33 [18.5%], 11 [9.2%], 3 [8.6%], *P* = 0.04); and CHADS2 score (1.1 ± 1.0, 0.8 ± 0.8, 0.8 ± 1.0, *P* = 0.007, respectively). AADs were prescribed after the RFCA during the blanking period, and there were no significant differences between the various classes of AADs regardless of the *HRC* SNP genotype. The results of the parameters from the transthoracic echocardiographic, transesophageal echocardiographic, and electrophysiological studies are summarized in Table 2. There was no significant difference in these study parameters between the PAF patients regardless of the *HRC* SNP genotypes.

**Table 1:** Baseline characteristics in each *HRC* SNP (rs3745297) genotype group.

	TT (wild type)	TG (hetero)	GG (homo)	P value
N	179	120	35	
Age (years)	64 ± 10	60 ± 12	59 ± 13	0.001
Male	136 (76%)	96 (80%)	24 (68.6%)	0.365
Duration (days)	1236 ± 134	1286 ± 163	1711 ± 302	0.353
BMI	23.8 ± 0.25	24.1 ± 0.3	23.8 ± 0.6	0.738
Hypertension	117 (66.1%)	60 (50%)	13 (37.1%)	0.001
Diabetes mellitus	33 (18.5%)	11 (9.2%)	3 (8.6%)	0.041
Family history	7 (4.1%)	2 (1.7%)	3 (8.6%)	0.173
Pre-history of heart failure	3 (1.7%)	1 (0.8%)	1 (2.9%)	0.667
Pre-history of stroke	14 (8.0%)	8 (6.8%)	2 (5.7%)	0.856
Alcohol	142 (79.5%)	103 (86.1%)	24 (68.2%)	0.153
Warfarin	154 (86.5%)	97 (80.8%)	27 (77.1%)	0.250
DOAC	25 (13.5%)	23 (19.2%)	8 (22.9%)	0.287
AAD				
Ia	45 (21.3%)	31 (25.8%)	4 (11.4%)	0.140
Ic	30 (16.9%)	14 (14.5%)	4 (11.4%)	0.405
II	57 (32%)	36 (30%)	10 (28.6%)	0.887
III	13 (7.3%)	6 (5%)	5 (14.3%)	0.217
Bepridil	24 (13.6%)	16 (13.3%)	9 (25.7%)	0.200
CHADS2 score	1.1±1.0	0.8±0.8	0.8±0.8	0.007
AHI	19.1±14.1	16.7±12.1	17.1±18.2	0.738

Values are the means ± SD or n (%), BMI = body mass index,

DOAC = direct oral anticoagulant, AAD = antiarrhythmic drug, AHI = apnea hypopnea index,

*HRC* = Histidine-rich calcium-binding protein, SNP = single nucleotide polymorphism

**Table 2:** Cardiac Parameters in each *HRC* SNP (rs3745297) genotype group.

	TT (wild type)	TG (hetero)	GG (homo)	P value
N	179	120	35	
TTE				
LAD	38.3 ± 6.0	37.8 ± 5.6	37.6 ± 6.1	0.687
LVEF	62.5 ± 5.3	61 ± 5.4	61.9 ± 5.0	0.062
LVDd	48.1 ± 4.6	48.6 ± 4.7	47.6 ± 5.1	0.478
LVDs	31.6 ± 3.9	32.4 ± 4.3	31.4 ± 6.7	0.208
IVS	9.0 ± 2.0	8.9 ± 1.4	8.6 ± 1.3	0.439
LAV	66.1 ± 19.7	63.2 ± 15.8	62.8 ± 15.3	0.363
LAVI	38.8 ± 11.3	36.8 ± 9.7	36.8 ± 8.4	0.346
TEE				
LAA area	457.1 ± 148.8	452.5 ± 144.2	441.3 ± 174.1	0.846
LAA flow	62.1 ± 18.5	58.4 ± 18.2	59.6 ± 20.2	0.229
EPS				
SNRT	1301.6 ± 315.8	1424.2 ± 787.7	1264.7 ± 235.7	0.118
cSNRT	465.7 ± 249.1	586 ± 745.3	444 ± 143.5	0.096
AH interval	94.0 ± 24.3	92.8 ± 22.3	93.7 ± 23.6	0.914
HV interval	41.2 ± 8.0	40.1 ± 10.0	41.7 ± 13.8	0.582
1:1 AVN	155.8 ± 25.3	155.0 ± 25.4	151.6 ± 17.5	0.684

Values are the means ± SD or n (%), TTE = transthoracic echocardiography, LAD = left atrial diameter, LVEF = left ventricular ejection fraction, LVDd = left ventricular diastolic diameter, LVDs = left ventricular systolic diameter, IVS = intraventricular septum, LAV = left atrial volume, LAVI = left atrial volume index, SNRT = sinus node recovery time, cSNRT = corrected SNRT, AH = atrium to His bundle, HV = His bundle to ventricle, AVN = atrioventricular node, *HRC* = Histidine-rich calcium-binding protein, SNP = single nucleotide polymorphism

### *HRC* SNP genotypes and AF recurrence

**Fig 1:**
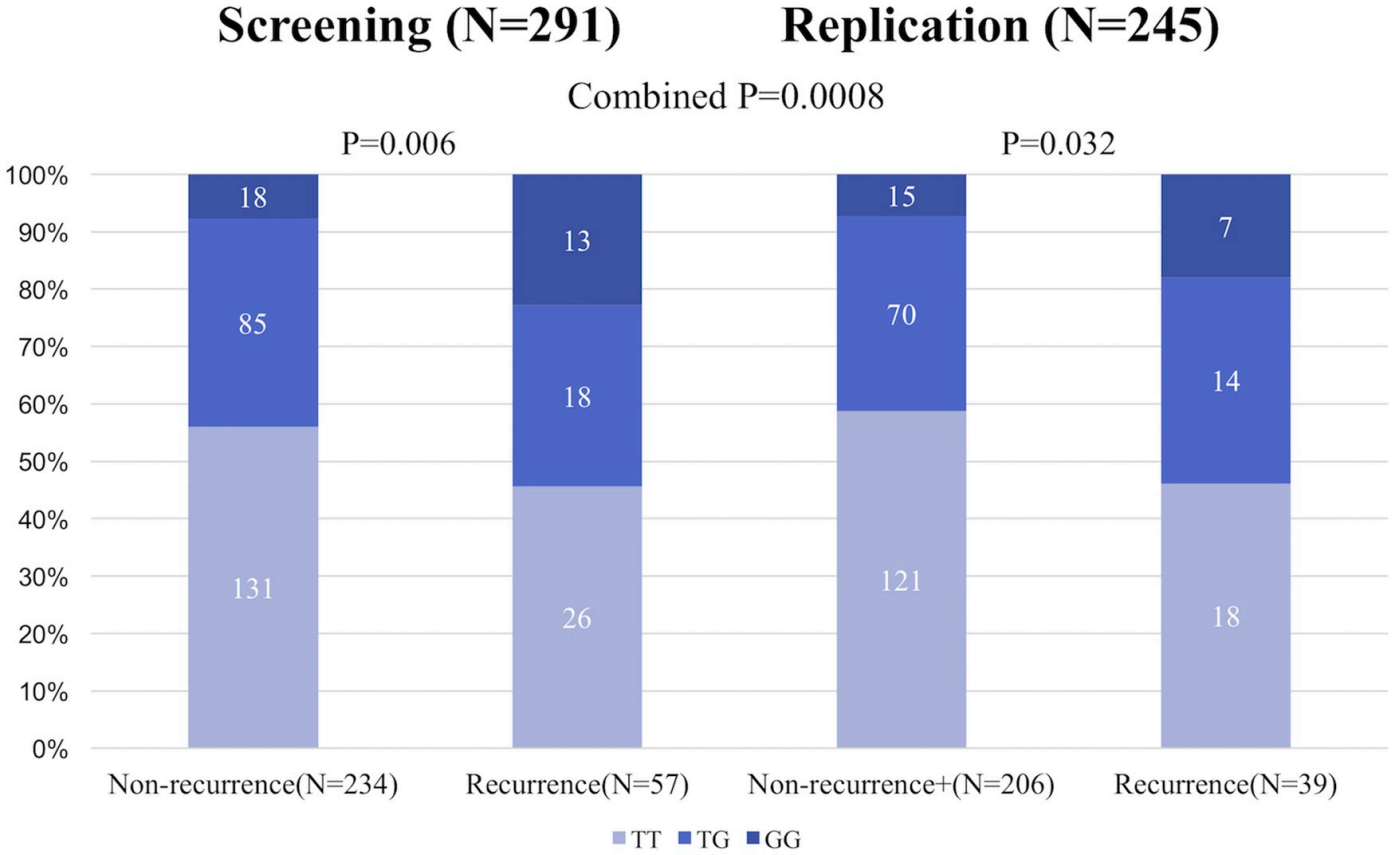
The minor G allele (Ser96Ala) rate was significantly higher in the PAF patients with AF recurrence than in those with AF non-recurrence in the screening set (allele frequency model OR, 1.80; *P* = 0.006) and replication set (allele frequency model OR, 1.74 [*P* = 0.032]).

**Fig 2:**
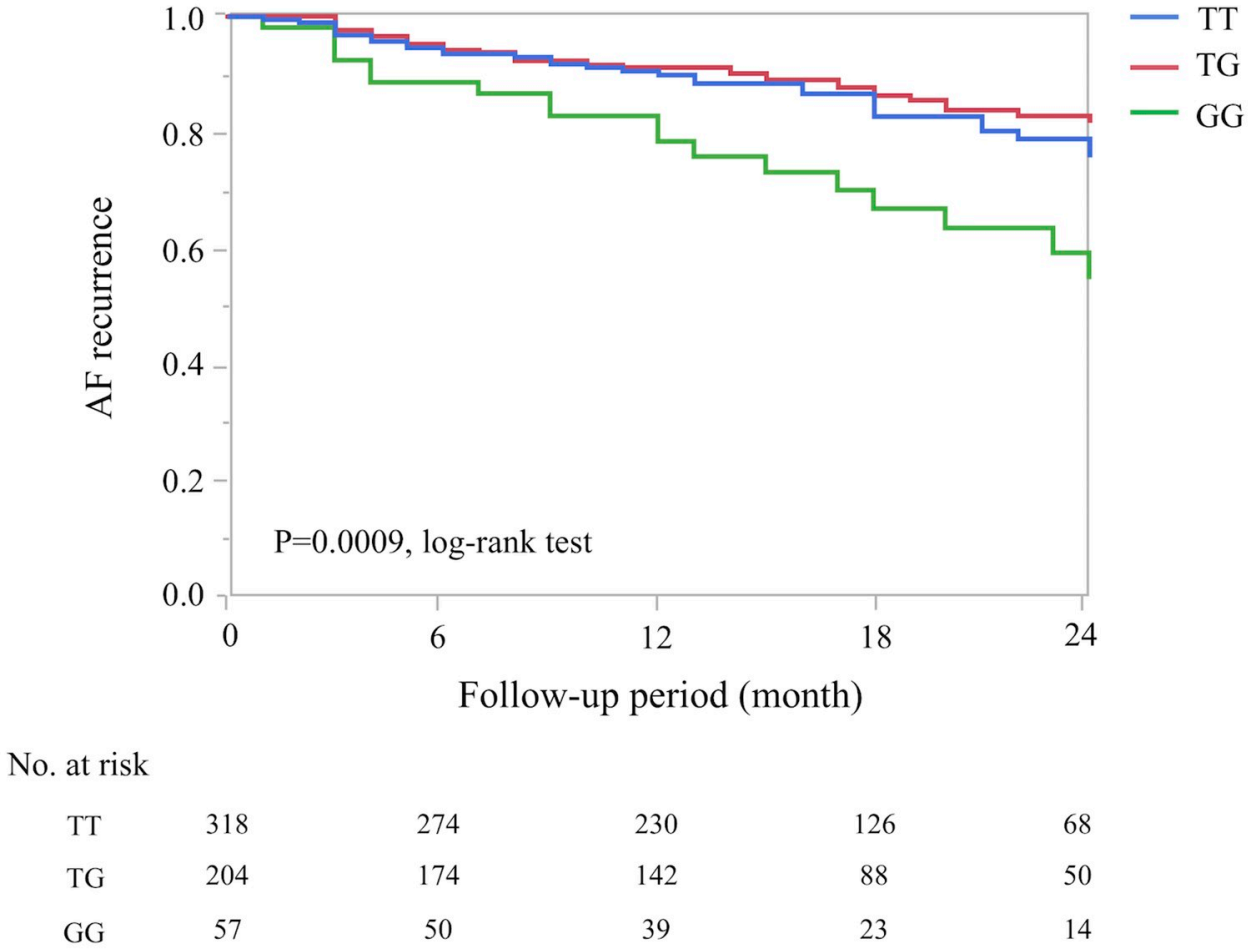
A Kaplan–Meier analysis of AF recurrence with the (*HRC*) SNP in each genotype group. PAF patients with a GG genotype (Ser96Ala homo type) were more likely to have recurrence than those with the other genotypes (*P* = 0.0004, log-rank test).

The genotype distributions of *HRC* SNP (Ser96Ala) in the screening and replication sets from patients with and without recurrences (AF non-recurrence) are summarized in Fig 1 and Table 3. During the 19 ± 9 months (range, 6 to 61 months) follow-up period, late AF recurrences were recognized in 57 patients in the screening set (i.e., AF non-recurrence, *N* = 277). The minor G allele (Ser96Ala) rate was significantly higher in the PAF patients with AF recurrence than in those with AF non-recurrence in the screening set (allele frequency model OR, 1.80; *P* = 0.006 and recessive model OR, 3.55; *P* = 0.0009). We also confirmed this significant association between the *HRC* Ser96Ala and AF recurrence in the replication set (15 ± 9 months follow-up period, AF recurrence vs. AF non-recurrence, *N* = 39 vs. *N* = 206; allele frequency model OR, 1.74 [*P* = 0.032] and recessive model OR, 2.79 [*P* = 0.032]; Fig 1 and Table 3). The Breslow-Day test showed no heterogeneity among the screening and replication groups, and the overall degree of association by the Mantel-Haenszel test was a *P* (allele) = 0.0008 (OR 1.78, 95%CI 1.28–2.48) (Table 3). A Kaplan–Meier analysis, which included both screening and replication (*N* = 579), revealed that AF recurrences were significantly higher in patients with a minor GG genotype (Ser96Ala homo) than in those with other genotypes during the follow-up period (*P* = 0.0009 log-rank test; Fig 2).

**Table 3:** The HRC SNP (rs3745297, Ser96Ala) genotype distribution in AF recurrence and AF non-recurrence groups both in the screening and replication sets.

		Genotype Distribution			HW Test	MAF	Allelic Model (T vs G)			Dominant Model			Recessive Model		
										(TG+GG vs TT)			(GG vs TG+TT)		
		TT	TG	GG	P		P^a^	P^b^	OR (95%CI)	P^a^	P^b^	OR (95%CI)	P^a^	P^b^	OR (95%CI)
**Screening**	**Non-recurrence**	131	85	18	0.12	0.26	0.006		1.80	0.159		1.51	0.0009		3.55
	**Recurrence**	26	18	13	0.42	0.39			(1.17–2.78)			(0.85–2.71)			(1.62–7.76)
**Replication**	**Non-recurrence**	121	70	15	0.28	0.24	0.032		1.74	0.146		1.66	0.032		2.79
	**Recurrence**	18	14	7	0.17	0.36			(1.04–2.92)			(0.84–3.30)			(1.05–7.36)
**Combined**^c^							0.0008	0.9273	1.78	0.0578	0.8432	1.57	0.0002	0.7048	3.22
									(1.28–2.48)			(1.01–2.45)			(1.75–5.93)

HRC: Histidine-rich Ca-binding protein, SNP: single nucleotide polymorphism, MAP: minor allele frequency

^a^ P value of chi-square test

^b^ Result of Breslow-Day test

^c^ Combined meta-analysis was performed using the Mantel-Haenszel method

Among the 57 patients with a late AF recurrence, only 16 (28.1%) underwent a second RFCA. Their AF trigger was from the PVs in nine patients, superior vena cava in one, septum in one, and unknown in five, respectively. Six patients (37.5%) had an *HRC* Ser96Ala, and there was no significant correlation between the AF trigger site and *HRC* genotype.

**Fig 3:**
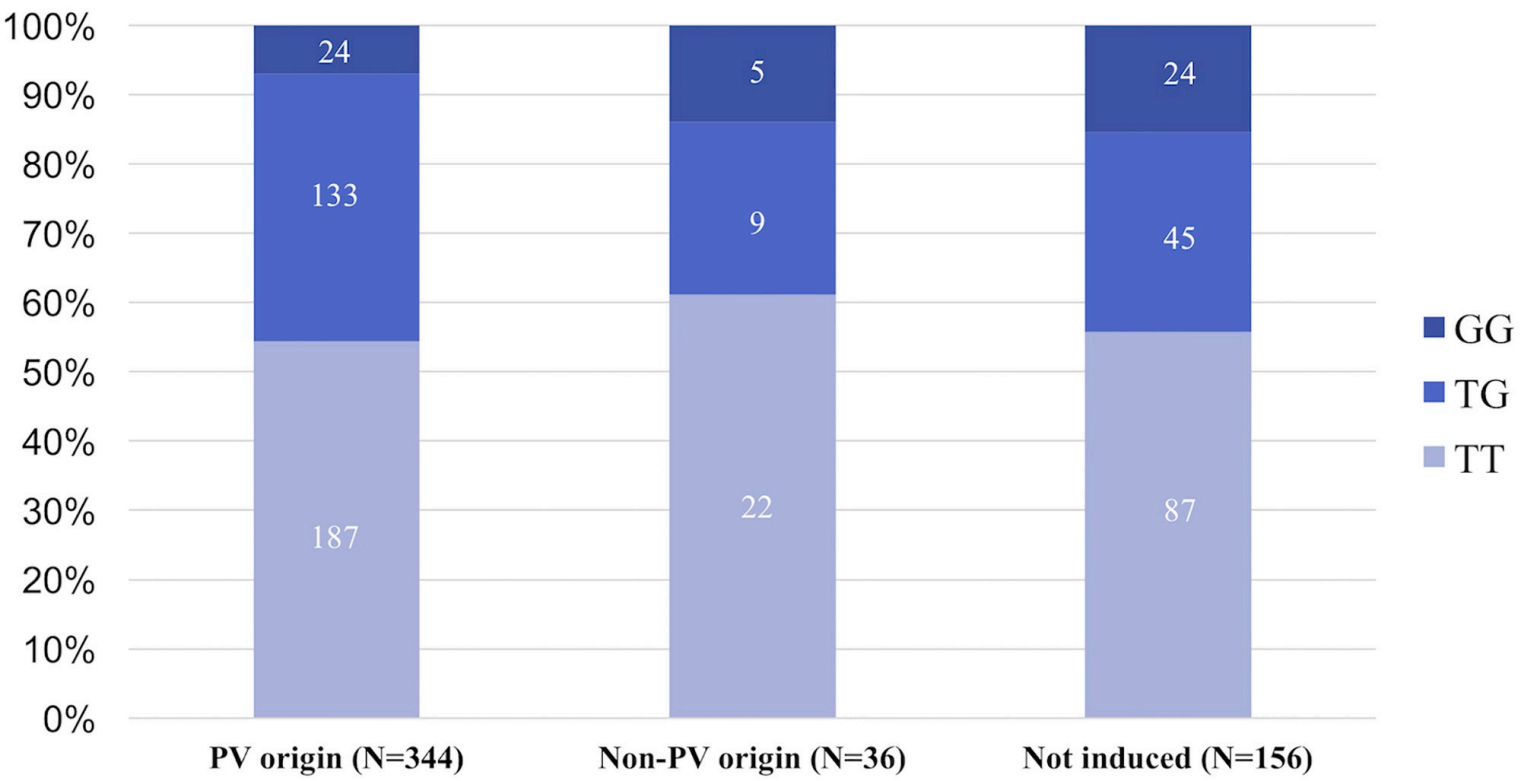
The minor allele frequency (MAF) of *HRC* SNP was similar in the PAF patients whose AF triggers were from a PV or non-PV origin (PV vs. non-PV, TT/TG/GG = 187/133/24 vs. 22/9/5, MAF 0.263 vs. 0.264, *P* = 0.98).

We defined a “PV origin” as when the AF was induced from the PVs after an ISP infusion and sinus rhythm was maintained after the initial AF ablation. We detected a PV origin in 344 PAF patients and non-PV origin in 36. No foci could be determined in the other patients before the EEPVI (not induced, N = 156). The sites of non-PV foci were the superior vena cava (*N* = 12), right atrial septum (*N* = 7), left atrial septum (*N* = 5), left atrial body (*N* = 2), and unknown (AF was evoked from non-PV foci, but the origin was not identified) or multiple (*N* = 10). The MAF of the *HRC* SNP was similar in the PAF patients whose AF triggers were from the PVs or non-PV origins (PV vs. non-PV, TT/TG/GG = 187/133/24 vs. 22/9/5, MAF 0.263 vs. 0.264, *P* = 0.98) (Fig 3).

### Multivariate analysis of AF recurrence

A multivariate analysis revealed that the duration of AF (time from the AF onset to the RFCA), sinus node dysfunction (sinus node recovery time >1.5 s), and *HRC* SNP Ser96Ala were independent predictors of AF recurrence (hazard ratio [HR], 95% CI, *P* value: 1.04, 1.00–1.08, *P* = 0.037; 2.42, 1.3–4.33, *P* = 0.018; and 2.66, 1.32–5.0, *P* = 0.007, respectively; Table 4).

**Table 4:** Univariate and multivariate analysis for the recurrence of atrial fibrillation.

Variables				Univariate			Mulitivariate	
	Recurrence	Non-recurrence	HR	95% CI	P value	HR	95% CI	P value
Age (years)	60±10	62±11	0.99	0.97–1.01	0.201			
Duration (per day)	2.5 [1.0, 7.1]	1.6 [0.5, 4.5]	1.05	1.01–1.08	0.030	1.0040	1.00–1.08	0.037
Hypertension	33 (57.9%)	157 (57.1%)	1.08	0.64–1.85	0.771			
Diabetes mellitus	8 (14.0%)	39 (14.1%)	0.84	0.37–1.67	0.637			
CHADS2 score	0.7±0.6	1.0±1.0	1.30	0.96–1.82	0.096	1.25	0.56–1.12	0.203
SNRT >1500ms	16 (30.7%)	44 (17.1%)	2.25	1.22–4.00	0.011	2.42	1.30–4.33	0.018
AH interval >140ms	1 (2.0%)	8 (3.2%)	0.97	0.06–4.47	0.980			
LAD >40mm	26 (45.6%)	95 (34.2%)	1.55	0.91–2.61	0.103	1.37	0.76–2.42	0.287
LAVI >40	16 (33.3%)	73 (28.9%)	1.33	0.71–2.39	0.359			
LAA flow <40 cm/s	5 (8.9%)	37 (13.6%)	1.36	0.6–1.68	0.498			
HRC (additive model)	13 (22.8%)	22 (7.9%)	2.85	1.47–5.14	0.003	2.66	1.35–5.00	0.007

Values are the means ± SD, n (%), [interquartile range] as appropriate, HR = hazard ratio, CI = confidence interval, SNRT = sinus node recovery time, AH = atrium to His bundle, LAD = left atrial diameter, LAVI = left atrial volume index, LAA = left atrial appendage, HRC = histidine-rich calcium-binding protein.

## Discussion

RFCA is an increasingly used treatment option for AF, but a certain number of patients suffer from recurrences. An investigation of the predictors of an AF recurrence is difficult because the patient population in which AF occurs is heterogeneous and whether to predict a recurrence depends on the patient factors as well as technical aspects of the procedure [1, 2]. The patient factors, such as the duration of AF, hypertension, diabetes, obesity, sleep apnea, alcohol intake, and sinus node dysfunction, are reported to be associated with AF recurrence, and a lifestyle modification is known to be important for the maintenance of sinus rhythm [2].

A genetic association with AF occurrence has been investigated actively using the genome-wide association study (GWAS). Overall, 14 genetic loci have been reported to be associated with AF in European and Asian ancestry groups, and 12 more new genetic variants associated with AF were reported in 2017 [24, 25]. The relationship between the AF-related SNPs reported in the GWAS analysis and AF recurrence after RFCA is gradually attracting attention, but it is still unclear. The results of the GWAS analysis revealed that AF-related 4q25 SNP near *PITX2* (rs2200733) increased the AF recurrence risk after RFCA [6, 26]. Husser et al. reported that only calcium signaling and extracellular matrix–receptor interaction pathways are associated with a late recurrence of AF after RFCA using an enrichment analysis of the GWAS data [27]. The relationship between the genetic factors and AF recurrence remains poorly understood.

Abnormal Ca^2+^ handling is known to evoke PV triggers, and the regulatory proteins of Ca^2+^ handling are known to be important contributors to an SR Ca^2+^ overload, diastolic membrane instability, and AF occurrence [8, 9]. A Ca^2+^ overload is a key mechanism of AF occurrence and maintenance, is linked to atrial structural and electrical remodeling, and influences AF maintenance [28]. *HRC* is also a modulator of the Ca^2+^ handling regulating the Ca^2+^ uptake through a direct interaction and inhibits the cardiomyocyte relaxation in the SR. The human genetic variant of *HRC*, rs3745297 (Ser96Ala), is a famous SNP that results in the abolishment of the *HRC* phosphorylation site by Fam20C kinase and dysregulation of the SR Ca^2+^ cycling. [13–15, 29].

We hypothesized that Ser96Ala was related to the recurrence of AF after RFCA in patients with PAF and investigated the relationship between Ser96Ala and AF recurrence. The main finding of this study was that the *HRC* genetic variant Ser96Ala is an independent predictor of PAF recurrence apart from the duration of AF and sinus node dysfunction. In our study, the minor (G) allele frequency of rs3745297 (Ser96Ala) was 28.4% in the screening of the PAF patients and 26.1% in the replication the PAF patients, which was consistent with the frequencies reported in the Hap Map Project (25.4% in East Asian populations). On another front, the frequency of Ser96Ala was higher in the PAF patients with an AF recurrence. Patients with PAF and the *HRC* genetic variant Ser96Ala were liable to have recurrent AF regardless of whether they were younger, and their rate of hypertension or diabetes was lower than that in those without Ser96Ala.

Hayashi et al. reported that patients with sick sinus syndrome (SSS) are at high risk for AF recurrence after RFCA [30]. They also noted that non-PV foci were found significantly more often to trigger AF in the SSS group, and AF and SSS are associated with electrical or structural remodeling. Another report indicated the relationship between the AF duration and recurrence after RFCA [31]. Takahashi et al. implied a relationship between the AF duration and extent of a fibrillatory substrate [32]. We first found that *HRC* Ser96Ala was associated independently with AF recurrence. The precise mechanism by which PAF patients with *HRC* Ser96Ala are likely to have a recurrence has not been clarified. The frequency of the *HRC* Ser96Ala was similar in the PAF patients with PV and non-PV origins. In a small number of AF recurrence cases undergoing a second RFCA, we did not find a relationship between the AF origin and *HRC* Ser96Ala. A recent meta-analysis revealed that more than half of the patients had at least one PV electrically reconnected even among AF-free patients and the PV reconnection did not always lead to AF recurrence [7]. *HRC* Ser96Ala may contribute to AF recurrence in PAF patients with a PV reconnection after the RFCA.

*KCNN2*, encoding the small-conductance Ca2+ activated K+ channel (SK2), which can shorten the cardiac action cardiac action potential duration, has been recognized as a new genetic locus in GWAS [25]. Given that *HRC* Ser96Ala causes an abnormal Ca^2+^ release, in AF patients with *HRC* Ser96Ala, their SK2 currents in the atrium may be easily activated and they are vulnerable to have a recurrence after the AF ablation.

Greiser et al. reported that an altered Ca^2+^ signaling during AF progression consists of three phases: Ca^2+^ overload, remodeling, and a steady state [33]. Similarly, after AF termination, three distinct phases of “recovery” of the intracellular Ca^2+^ handling occur: calcium unloading, reverse remodeling, and full recovery. If *HRC* Ser96Ala exists in PAF patients, they may be exposed continuously to a Ca^2+^ overload and may not be able to obtain a full recovery. The Ca^2+^ loading leads to I_CaL_ (Ca^2+^) channel inactivation, reducing the action potential duration, causing atrial structural and electrical remodeling, which may set off an AF trigger in both the PV and non-PV areas, falling into a vicious cycle [34]. These processes may be the reason why PAF patients with the *HRC* Ser96Ala genetic variant are prone to have AF recurrences.

The present study had several limitations. First, it was a retrospective study conducted at a single center, and the total subjects and number of recurrence cases were small. Second, we did not reveal the mechanism by which the *HRC* genetic variant influences AF recurrence. AF recurrence results from many factors, so we need to show evidence of its mechanism with an animal experiment or in silico. We identified a clinical implication that the *HRC* SNP Ser96Ala may be a promising genetic marker of AF recurrence and useful to determine an adequate therapeutic strategy. Therefore, we must validate the association between AF recurrence and the *HRC* SNP Ser96Ala in a larger sample of cases and controls.
